# Immobilization of Diatom *Phaeodactylum tricornutum* with Filamentous Fungi and Its Kinetics

**DOI:** 10.4014/jmb.2209.09042

**Published:** 2022-11-21

**Authors:** Tyler J. Barzee, Hamed M. El-Mashad, Andrew R. Burch, Annaliese K. Franz, Ruihong Zhang

**Affiliations:** 1 Department of Biosystems and Agricultural Engineering, University of Kentucky, Lexington, KY 40546, USA; 2 Department of Biological and Agricultural Engineering, University of California Davis, Davis, CA 95616, USA; 3 Agricultural Engineering Department, Mansoura University, El Mansoura, Egypt; 4 Department of Chemistry, University of California, Davis, CA 95616, USA; 5 Biochemistry, Molecular, Cellular and Developmental Biology Graduate Group, University of California, Davis, CA 95616, USA

**Keywords:** Microalgae immobilization, fungi, *Aspergillus* sp., *Phaeodactylum tricornutum*, heat-deactivation, Freundlich isotherm

## Abstract

Immobilizing microalgae cells in a hyphal matrix can simplify harvest while producing novel mycoalgae products with potential food, feed, biomaterial, and renewable energy applications; however, limited quantitative information to describe the process and its applicability under various conditions leads to difficulties in comparing across studies and scaling-up. Here, we demonstrate the immobilization of both active and heat-deactivated marine diatom *Phaeodactylum tricornutum* (UTEX 466) using different loadings of fungal pellets (*Aspergillus* sp.) and model the process through kinetics and equilibrium models. Active *P. tricornutum* cells were not required for the fungal-assisted immobilization process and the fungal isolate was able to immobilize more than its original mass of microalgae. The Freundlich isotherm model adequately described the equilibrium immobilization characteristics and indicated increased normalized algae immobilization (g algae removed/g fungi loaded) under low fungal pellet loadings. The kinetics of algae immobilization by the fungal pellets were found to be adequately modeled using both a pseudo-second order model and a model previously developed for fungal-assisted algae immobilization. These results provide new insights into the behavior and potential applications of fungal-assisted algae immobilization.

## Introduction

Microalgae cultivation is an industrially relevant strategy for the production of a wide variety of biological products including food, feed, nutraceuticals, and biofuel, and offers a valuable unit operation for wastewater treatment [1]. Immobilization of microalgal cells to biotic or abiotic support materials (*i.e.*, scaffolds) has been investigated for intensified microalgae cultivation and wastewater treatment as well as for the production of engineered biocomposites with a wide range of potential applications [2, 3]. Scaffolds derived from a biological origin (*e.g.*, alginate, chitosan, gelatin, fungal biomass, latex, marine sponge) are of particular interest due to their compatibility with food/feed [4] and biomedical applications [5]. The ability of filamentous fungi to immobilize microalgae cells in a process mimicking lichen formation has attracted substantial interest in recent years [6-16] with potential advantages including synergistic growth and preferred nutrient composition of the resulting biomass [17]. Several applications of fungal-assisted algae immobilization have been explored such as production of edible food ingredients [4, 18], wastewater treatment [11, 19, 20], biofuel production [11, 21], biogas purification [20], and bioremediation of pharmaceuticals [22]. Examples of potential future applications of microalgae immobilization include synergistic coculturing, design of new cultivation bioprocesses, and additive manufacturing of novel myco-algae materials. Despite the promise of fungal-assisted algae immobilization, more development is needed on modeling and optimizing the process as well as increasing the breadth of microalgae species investigated. For instance, the marine diatom *Phaeodactylum tricornutum* is an industrially relevant microalgae species with publicly-accessible genomic information [23, 24] that has been proposed as a candidate for biofuel and bio-oil production [25-27] but no information has been reported thus far on its behavior during fungal-assisted immobilization.

The mechanism(s) of the biosorption process involved in fungal-assisted immobilization are complex and poorly understood although recent reports have identified a potential role of fungal extracellular polymeric substances (EPS) and specifically *N*-acetyl-glucosamine-mediated interactions with microalgae cells [16]. This proposed mechanism involves induced microalgae surface changes that mediate attachment to fungal hyphae. It has been reported that viable fungal biomass is a requirement of or aids the immobilization process [13, 16] but it is not yet clear if viable microalgae cells are similarly necessary. In addition to the important implications this question has for a mechanistic understanding of the process, it is also relevant to industrial applications of this technology since partially- or fully-inactive microalgae cultures are common in industrial microalgae production operations due to purposeful deactivation (*e.g.*, pasteurization), culture crashes from biotic (*e.g.*, contamination with competing microorganisms) or abiotic factors (*e.g.*, unexpected changes in temperature, other environmental parameters, and/or reactor chemistry), or extended storage of previously active cultures. In addition, microalgae immobilization is of interest for many other reasons besides simple harvesting, in which cases active microalgae may not be desired and/or interfere with subsequent processes. For instance, deactivated microalgae might be preferred for processes involving secondary fungal cultivation steps where the mixotrophic growth capabilities of many microalgae would otherwise create competition for organic carbon/other nutrients and potentially lead to inconsistencies in the final product composition. Mathematical modeling of the immobilization capacity and kinetics of the fungal-assisted immobilization process lends insight into its mechanisms and is necessary for performance prediction in industrial applications. Bhattacharya *et al*. [15] proposed a mathematical model explaining the growth of fungal-algal pellets during the immobilization process as a function of the pellet geometry and velocity profile of the medium. Physical adsorption models (*e.g.*, Freundlich isotherm) typically applied to the removal of contaminants from water and wastewater may reveal further insights into the immobilization behavior and allow prediction of the required loading of fungi for effective removal of microalgae cells, a critical design parameter of scaled-up applications.

This study presents one of the first examples of fungal-assisted immobilization of *P. tricornutum* and of the effect of microalgae activity state (*i.e.*, active vs heat-deactivated) on its immobilization potential. The study also presents the first systematic application of mathematical kinetic and equilibrium models to the fungal-assisted microalgae immobilization process. The specific objectives of this study were to: 1) investigate the effect of heat-deactivation on the immobilization efficiency of *P. tricornutum* with *Aspergillus* sp. fungus pellets; and 2) determine the kinetics of immobilization and demonstrate the application of adsorption isotherm models to the fungal-assisted algae immobilization process.

## Materials and Methods

### Microalgae Cultivation

*Phaeodactylum tricornutum* microalga (UTEX 466) was cultivated using methods previously published [26, 28]. Briefly, a culture was cultivated in 2-L Corning Pyrex media bottles (Corning 1395-2L) on f/2 media (NCMA, USA) at 23°C with constant stirring, filtered air bubbling, and full spectrum lighting (T-5 grow lights) on a 16:8 light/dark cycle (60-120 μmol photons/m^2^/s). Microalgae growth was monitored by absorbance measurements at 680 nm (ABS_680_) and the biomass was removed in late-exponential/early stationary phase for the fungal-assisted immobilization experiments. The microalgae biomass was quantified as volatile suspended solids (VSS) utilizing Standard Methods [29].

### Fungus Cultivation

Filamentous fungus *Aspergillus* sp. UCD F01, isolated from anaerobic digestate stored in the local environment, was used in the study. The isolate’s growth on solid PDA media was characterized by a single circular region of radial flat white growth that became covered in a black mat of surface spores at maturity. Microscopic observation of the isolate showed the presence of filamentous hyphal growth with the presence of septum and conidial structures. For identification, two petri dishes of mature fungal growth on PDA were shipped overnight on ice to EMSL Analytical labs (USA), where they were identified according to EMSL Method MICRO-SOP-202.

The spores from the fungal isolate grown on PDA were aseptically isolated by washing with 0.22 μm filtered water (MilliporeSigma 0.22 μm syringe filter) and counting the resulting spore suspension by a hemocytometer [19]. The spores were inoculated to autoclaved bottles each filled with 200 ml of potato dextrose broth (PDB) to a level of 1 × 10^4^ spores/ml of final solution. The bottles were then aerated with filtered air at 2 L/(L min) and incubated at 30°C for three days. The pellets were isolated from the solution by straining through cheese cloth. The diameter of the pellets was measured using calipers of at least 50 randomly selected pellets. The fungal biomass utilized in the immobilization experiments was quantified as volatile solids (VS) utilizing standard methods [29].

### Comparison of Fungal-Assisted Immobilization of Active and Heat-Deactivated *P. tricornutum* Microalgae

To test the hypothesis that viable algal biomass was required for the fungal immobilization process, this study investigated heat-treatment of algal biomass to deactivate the cells prior to loading the fungal pellets. A stock solution of *P. tricornutum* (0.406 g VSS/L) was heated to 75°C and held for 15 minutes and allowed to cool below 35°C before transferring 125 ml to glass 250 ml-capacity flasks. The temperature and duration of heat treatment were chosen due to their reported effectiveness in microalgae deactivation (<1% cell viability [30]) while limiting more dramatic chemical changes associated with higher temperature treatments [31]. The heat-treated (deactivated) algae biomass was visibly different from the stock with the color changing from brown to green, probably due to the loss of brownish pigments such as carotenoids under elevated temperatures [32]. The cooled deactivated *P. tricornutum* stock solution (0.377 g VSS/L) was divided in 125 ml increments to 250 ml-capacity flasks and four wet fungal pellets (~ 0.5 g biomass (w.b.), diameter of 7.86 ± 2.6 mm) were loaded to each flask. The flasks were then incubated at 30°C and shaken at 150 rpm (Benchmark Scientific Incu-Shaker 10L) with supplemental lighting (16:8 light/dark cycle, 24-28 μmol photons/m^2^/s) [19]. Suspended algae concentration was monitored by ABS_680_ of broth supernatant measured periodically over an approximately 5-day immobilization period. The percent of VSS removal was estimated as the reduction of ABS_680_ from each time point compared to the value at the initial time point [16]. A control experiment was simultaneously conducted using the active *P. tricornutum* stock. The experiments were carried out in duplicate and the average and standard deviations of experimental data are presented. Microscopic imaging and analysis were performed utilizing previously published methods [28].

### Kinetics of Fungal-Assisted Immobilization of Heat-Deactivated *P. tricornutum* Microalgae

Following the observation that heat-deactivated *P. tricornutum* was more favorably immobilized by the fungus than active *P. tricornutum*, an experiment was then carried out to study the immobilization kinetics and equilibrium behavior of the fungal-assisted algae immobilization process on heat-deactivated *P. tricornutum* microalgae. A stock solution of heat-deactivated *P. tricornutum* (0.518 g VSS/L) was prepared and divided in 125 mL increments to 250-mL flasks. Wet fungal pellets (diameter of 6.06 ± 0.69 mm) were loaded to the flasks to achieve average loadings of 0.28, 0.64, 1.30, and 2.53 g fungi VS/g algae VSS.

The flasks were then subjected to the same temperature, agitation, and lighting conditions as the previous experiment. Although the microalgae were deactivated by the heat treatment, light was provided to maintain consistency with other experiments from the literature [19]. ABS_680_ and pH measurements of supernatant were taken periodically over an approximately 3-day immobilization period. At the end of the experiment, representative samples of the supernatant from each flask were analyzed for TSS and VSS. The absorbance and VSS measurements were related through a calibration curve (VSS($\frac{mg}{L}$) = 544.6(*ABS*_680_) + 149.93, R^2^ = 0.87) that allowed for calculation of algae concentration at all time points. Although the determination coefficient is relatively high, small debris such as fungal pellet fragments or flocs of cells that developed during the immobilization process and slightly influenced absorbance measurements of well-mixed cuvettes.

The equilibrium immobilization capacity (*q_e_*, g algae removed/g fungi loaded) was calculated as:

$$q_{e}=\frac{(A_{i}-A_{e})*V}{F_{i}}$$ (1)

where *q_e_* is the equilibrium immobilization capacity of adsorbate (microalgae) by the adsorbent (fungi), *A_i_* is the equilibrium concentration of algae (g VSS/L) determined from the negative control (fungal loading of 0 g/g), *A_e_* is the equilibrium concentration of algae (g VSS/L) in the supernatant of each experimental flask, *V* is the reactor volume (0.125 L), and *F_i_* is the mass of fungi loaded to each reactor (g VS). The experiment was carried out in duplicate and the average and standard deviations of experimental data are presented.

### Adsorption Isotherm and Kinetics Modeling

The Freundlich isotherm model was fit to the experimental data and three kinetic models were investigated for their applicability to the immobilization process. The Freundlich isotherm is an empirical model capable of describing mono- and multi-layer adsorption onto homogenous and heterogeneous surfaces. The Freundlich isotherm model is [33]:

$$q_{e}=k_{f}\cdot C_{e}^{\frac{1}{n}}$$ (2)

where *k_f_* (g/g) (L/g)^1/n^ and *n* (dimensionless) are Freundlich constants and *C_e_* (g/L) is the concentration of adsorbate at equilibrium. The Freundlich isotherm constants can be determined through linear and nonlinear regression methods. The Freundlich isotherm model is linearized as:

$$\ln(q_{e})=\ln(k_{f})+\frac{1}{n}\ln(C_{e})$$ (3)

where a plot of *ln*(*q_e_*) and *ln*(*C_e_*) and the slope and intercept of the resulting regression line can be used to estimate the Freundlich constants.

The kinetics of immobilization of algae to fungus were quantified through pseudo first- and second-order models commonly utilized for physical adsorption [34, 35]. The pseudo first-order model is expressed mathematically as [36]:

$$\frac{dq_{t}}{dt}=k(q_{e}-q_{t})$$ (4)

where *q_t_* and *q_e_* are the immobilization capacities (g algae/g fungi) at time *t* (d) and equilibrium, respectively and *k* (d^-1^) is the pseudo-first order rate constant. The rate constant can be determined by plotting the linearized form:

$$\log(q_{e}-q_{t})=\log(q_{e})-\frac{k}{2.303}t$$ (5)

The pseudo first-order model could not be appropriately applied to the model because of discontinuities caused by some data points where the measured immobilization capacity at time *t* (q_t_) was greater than that at equilibrium (q_e_). Due to this discontinuity, a pseudo second-order model was utilized, which is expressed mathematically as [35]:

$$\frac{dq_{t}}{dt}=k{\left(q_{e}-q_{t}\right)}^{2}$$ (6)

where *k* (g/(g d)) is the pseudo-second order rate constant. In a similar manner as before, the rate constant can be determined by plotting the linearized form:

$$\frac{t}{q_{t}}=\frac{1}{kq_{e}^{2}}+\frac{t}{q_{e}}$$ (7)

An additional mathematical model proposed by Bhattacharya *et al*. [15] to describe the flocculation of live microalgae with fungal pellets was also utilized in this study. The rate of algal attachment to the pellet is assumed to occur due to physical contact between the pellet and the algal cells in suspension and an “attachment constant” *K_s_* is added to the model to describe the effects of shear stress or other factors that might hinder the immobilization process. The model notably excludes the contributions of fungal growth to the increasing size and mass of the pellet as well as potential loss of algal mass in the pellet matrix by consumption and degradation by the fungi. The model is expressed mathematically as:

$$HI=K_{s}(1-e^{-\lambda t^{3}})$$ (8)

where *HI* is the harvesting index defined as the proportion of algae biomass removed from solution (ranges from 0 to 1); λ (1/d^3^) is a lumped parameter that describes the velocity field characteristics, the geometry of the pellet, and certain other properties of the pellet such as its density; and *t* is time (d).

For the adsorption isotherm and pseudo-first and second order kinetics modeling, Microsoft Excel was utilized to perform linear regression. For the kinetics modelling using the Bhattacharya model, the Matlab curve fitting tool for the nonlinear regression model was used for determining the model coefficients and performing statistical analysis.

## Results

### Comparison of Fungal-Assisted Immobilization of Active and Heat-Deactivated *P. tricornutum* Microalgae

**Fig. 1:**
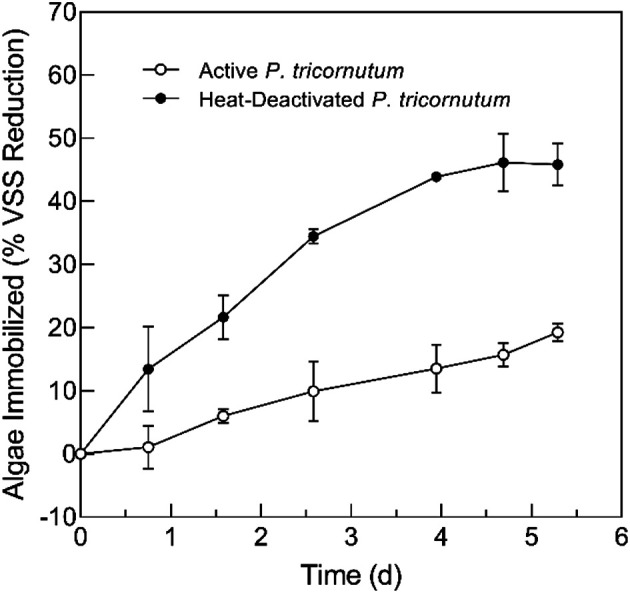
Fungal-assisted immobilization of active and heat-deactivated *P. tricornutum* microalgae by *Aspergillus* sp. UCD F01 fungal pellets. Data are presented as average ± standard deviation (*n* = 2).

During the immobilization of active and heat-deactivated *P. tricornutum* by *Aspergillus* sp. UCD F01, both solutions became visibly clearer as time progressed with visible growth and color change of the fungal pellets’ surfaces. Maximum average removals of *P. tricornutum* from solution were 19% and 46% for active and heat-deactivated microalgae, respectively (Fig. 1).

### Effect of Fungal Loadings on Removal of the Heat-Deactivated *P. tricornutum* Microalgae

**Fig. 2:**
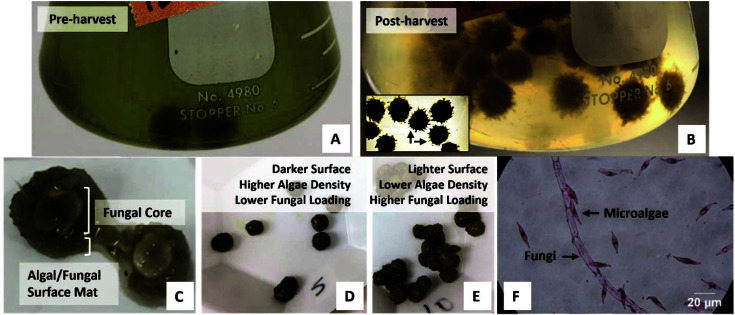
(**A**) Beginning of the immobilization process with a flask of heat-deactivated *P. tricornutum* and the addition of *Aspergillus* sp. UCD F01 fungal pellets (dark spheres); (**B**) the end of the immobilization process, subset arrows show filamentous hair-like structures; (**C**) a cross section of a fungal-algal pellet postimmobilization; post-immobilization pellets at a loading of (**D**) 0.73 g fungi/g algae and (**E**) 2.9 g/g; and (**F**) microscopic image (100× magnification) of the mat isolated from the surface of the pellet post-immobilization (sample stained with safranin to aid in contrast).

**Fig. 3:**
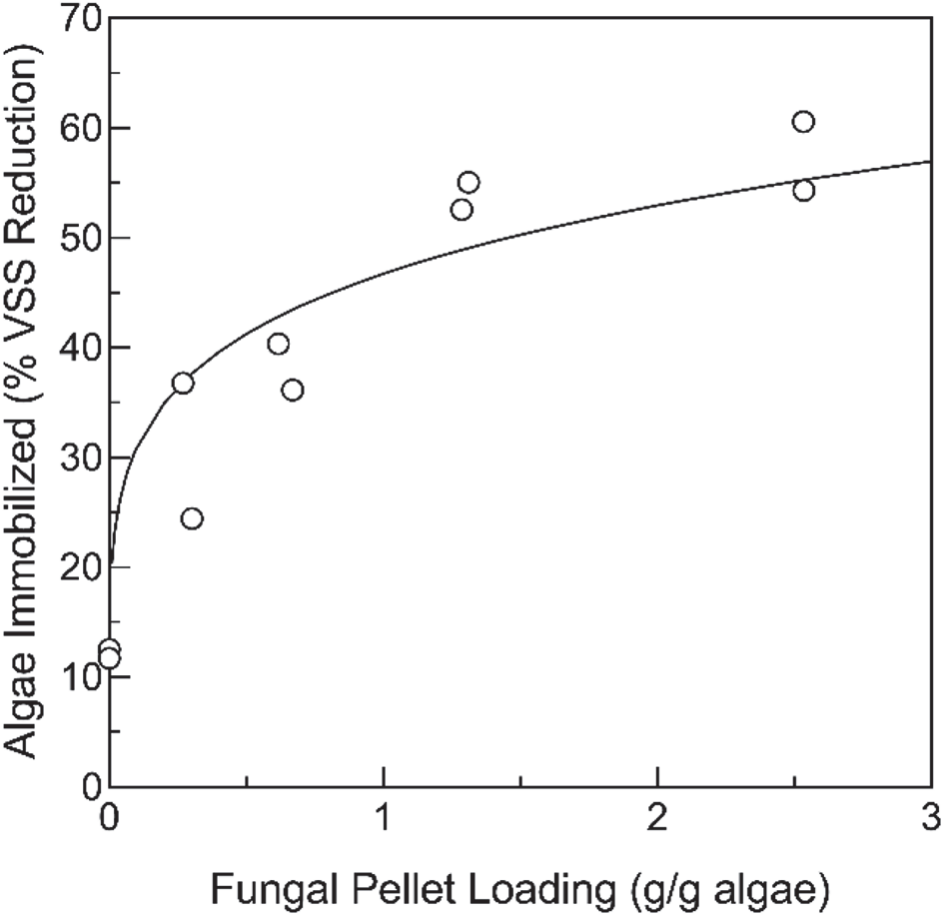
Immobilization efficiency based on percentage removal of heat-deactivated microalgae *P. tricornutum* from solution at different initial loadings of *Aspergillus* sp. UCD F01 fungal pellets.

Similarly to the previous experiment, over the course of the immobilization experiment with varying loadings of fungal pellets, the solution became visibly clearer over time due to the algae immobilization by the fungal pellets (Figs. 2A and 2B). The fungal pellets grew visibly larger with multiple hairy filaments developing on the pellet surface (Fig. 2B). A cross section of the pellets revealed a thick mat of microalgae that covered the fungal pellet (Fig. 2C). Microscopic observation of the fungus-algae mat from the surface revealed evidence of microalgae adhering to the fungal surfaces (Fig. 2F). The fungal pellets obtained at the end of the experiment from flasks with relatively low fungal loadings (0.73 g/g) were visibly darker in color than the pellets obtained from flasks with relatively high fungal loadings (2.9 g/g) (Figs. 2D and 2E). This was likely due to the high immobilization density of microalgae on the lower-loaded pellets, causing a darkening of the pellet surface. Higher fungal loadings led to increased algae immobilization (Fig. 3). Over the course of the 3-day experiment, 25%-60% of the microalgae was removed from the solution by fungal loadings of up to 2.5 g VS fungi/g VSS microalgae. The removal of heat-treated microalgae was in the range of that expected from the previous results.

### Adsorption Isotherm

**Fig. 4:**
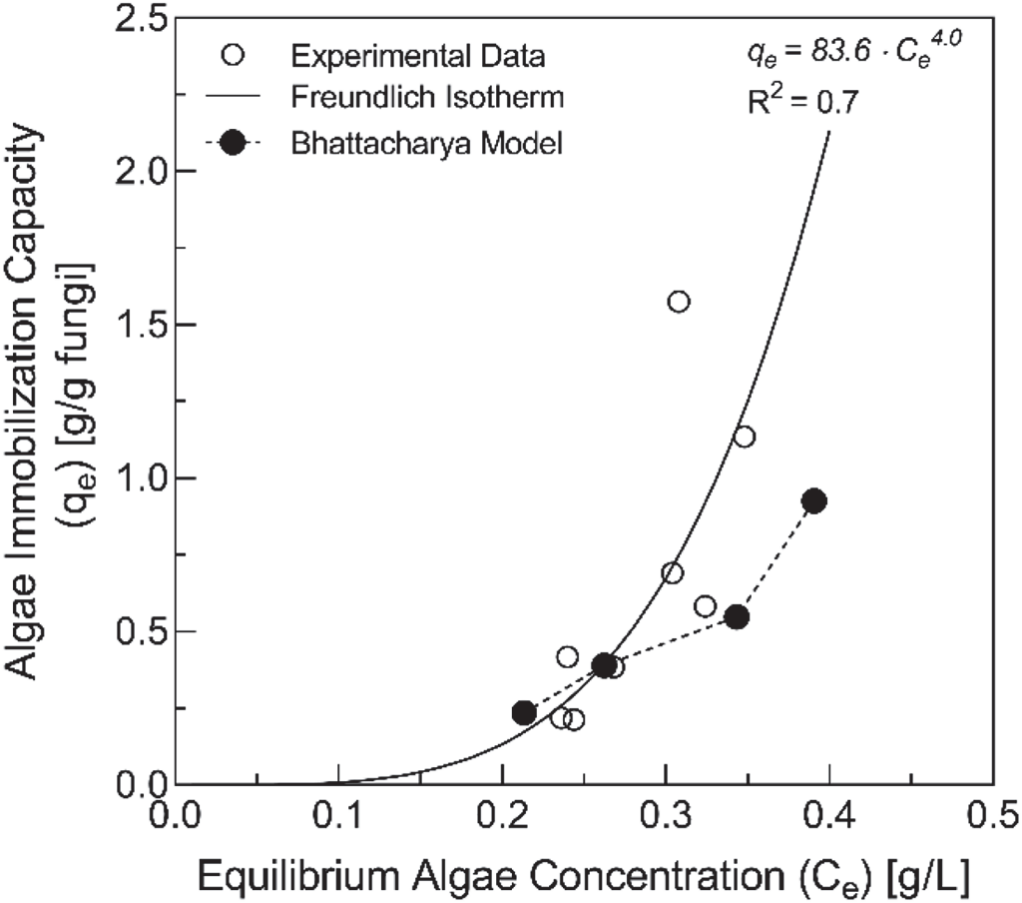
The Freundlich isotherm and Bhattacharya models applied to the immobilization of *P. tricornutum* by *Aspergillus* sp. UCD F01.

The equilibrium experimental data from Section 3.2 was fit to the Freundlich adsorption isotherm model (Fig. 4). In this experiment, a maximum average immobilization capacity of 1.57 g algae/g fungi was obtained, which indicated that the fungal isolate was able to remove more than its original mass of microalgae. It is important to note that the total mass of fungus at the end of the experiment was likely different than its initial mass due to cell growth and/or death during the immobilization process. The Freundlich constants *k_f_* and *1/n* were determined to be 83.6 and 4.0, respectively. The regression model was able to explain 70% of the variation in the data, indicating a moderate model fit. Estimates of K_s_ from Bhattacharya’s model (Table 1) were also used to calculate modeled equilibrium algae concentrations (*C_e_*) and immobilization capacity (*q_e_*) and plotted along with the Freundlich isotherm results in Fig. 4. The modeled estimates show similar behavior as the experimental data with a slightly less intense slope as the Freundlich isotherm model.

### Immobilization Kinetics

**Fig. 5:**
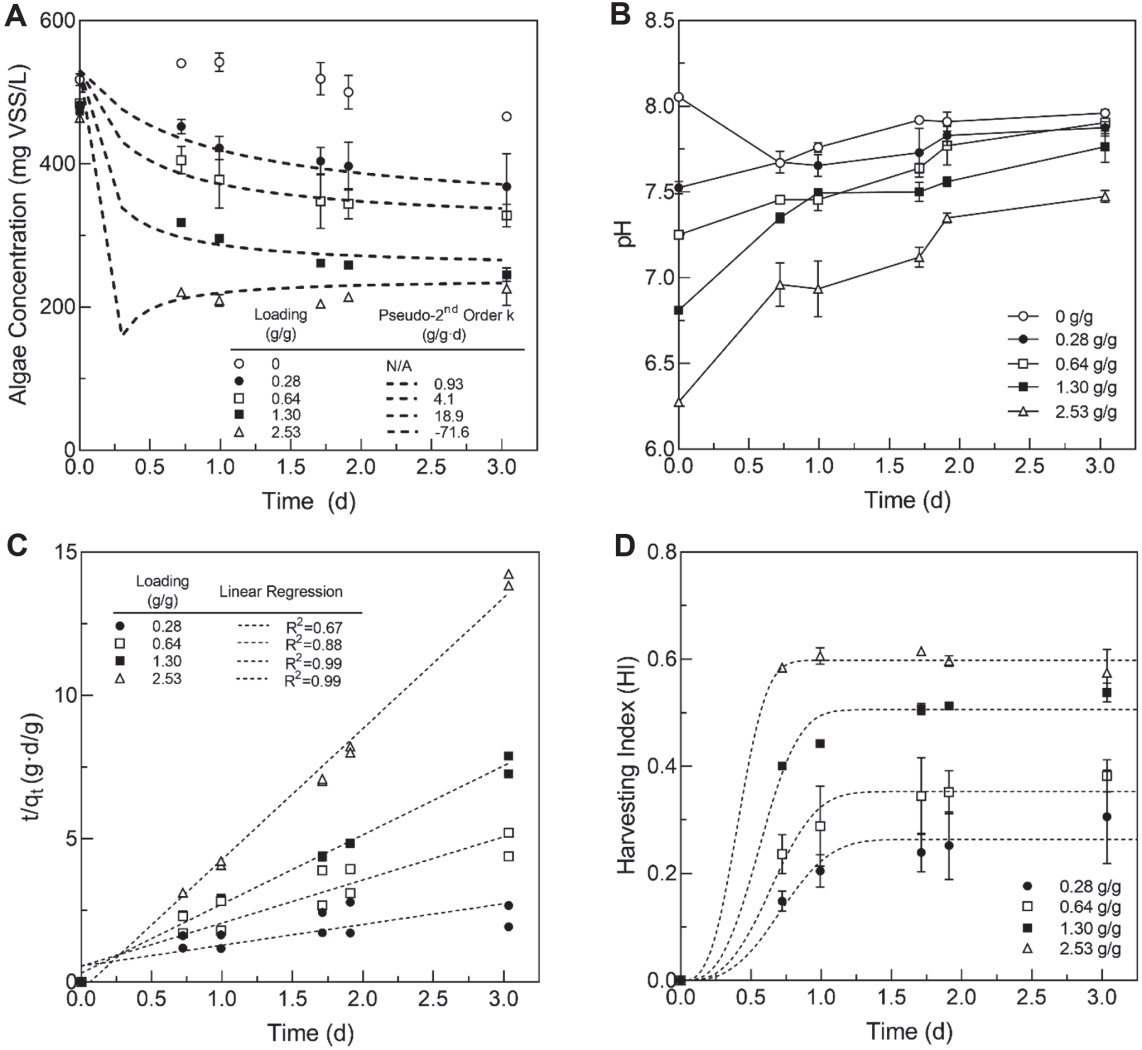
(**A**) Heat-deactivated *P. tricornutum* microalgae concentrations over time in response to different initial fungal (*Aspergillus* sp. UCD F01) pellet loadings along with the modeled fit to the pseudo-second order kinetic model, (**B**) the pH profile of the various fungal loadings in the experiment, (**C**) the plot used to determine the k constant in the pseudo-second order kinetic model, and (**D**) the Immobilization Index of the microalgae cultures over time and fit with the Bhattacharya kinetic model. Data are presented as average ± standard deviation (*n* = 2).

The time-series data for the reduction in algal biomass and the change in pH during the experiment can be seen in Figs. 5A and 5B. The rate and total amount of microalgae removed during the experiment increased with increased fungal loading. The initial pH of the medium was strongly influenced by the initial fungal loading with higher loadings leading to decreased pH, likely due to the low pH of the fungal growth medium. Fungal pellets were not rinsed prior to addition to microalgae cultures since this has been shown to inhibit or entirely prevent fungal assisted microalgae immobilization in a previous study [16]. The pH increased during the course of the experiment in all of the experimental flasks with pellet loadings. The pseudo second-order rate constant (*k*) of immobilization was determined to range between 0.94 and 18.9 g/(g d) for all except the highest fungal loading rate (Fig. 5A, Table 1), which displayed a non-physical negative *k* value from the plot of *t/q_t_* vs *t* (Fig. 5C). The pseudo second-order model was able to explain 67%-99% of the variation in the experimental data (Fig. 5C), indicating that it might be a generally appropriate model to explain the immobilization process. The Bhattacharya model was also applied to explore the kinetics of algae immobilization (Fig. 5D). The attachment coefficient (K_s_) varied with different fungal pellet loadings (Table 1) and can be essentially interpreted as the maximum proportion of the initial algae mass that was removed by the fungal pellets. This model was able to explain 84%-99% of the variation in the data, indicating a stronger model fit than the pseudo second-order model.

## Discussion

### Immobilization Efficiency and Effect of Microalgae Activity State

The immobilization efficiency of active *P. tricornutum* (19%) observed in this study was lower than the reported immobilization efficiency (~60%) of active *Nannochloropsis oceanica*, another marine diatom, by the fungus *Mortierella elongate*, although a lower immobilization efficiency (~25%) of *N. oceanica* was obtained using the fungus *Mortierella gamsii* [37]. This finding is consistent with other studies that have identified species-dependent relationships in the immobilization efficiencies where certain microalgae, fungi, or combinations thereof present better immobilization performance than others [11].

Our results indicate that active microalgae biomass is not a requirement for the immobilization of *P. tricornutum* by *Aspergillus* sp. UCD F01. While the active *P. tricornutum* was not immobilized effectively by the fungus in the present study (~20%), the heat-deactivated form presented high immobilization performance, up to 60%. This result is not consistent with the previously proposed mechanism [16] in which morphological changes (cell elongation and development of surface projections, presumed to be dependent on active microalgae) in the microalgae cells were induced by the attachment of *N*-acetyl glucosamine produced and released to solution by the fungal cells. The associated increase in the surface roughness of the microalgae cells is thought to be a critical component of the cellular attachment process [16, 38]. Heat treatment of microalgae cells, as performed in the present study, is known to cause irreversible damage to the cell structure including cell shrinkage, degradation of photosynthetic pigments, and loss of membrane integrity [39]. In light of the important role that surface roughness plays in the fungal immobilization process, it is possible that certain morphological changes following the heat treatment of *P. tricornutum* played a role in the increased immobilization efficiency of heat-treated microalgae cells observed here. While the applicability of heat treatment to industrial cultures of *P. tricornutum* as a means to increase immobilization efficiency are unlikely due to its associated expense, the results of this study are relevant to systems with partially- or fully-deactivated *P. tricornutum* cultures or that utilize pasteurization for other purposes such as for ensuring food safety or as a pretreatment to chemical extraction or other biological processes. Future research could be focused on the investigation of the effect of other microalgae deactivation methods or storage conditions on the performance of fungal-assisted algae immobilization.

### Adsorption Isotherm and Kinetics

For the adsorption isotherm, the positive value of *1/n* (4.0) suggests multi-layer immobilization as evidenced by the high levels of microalgae removed with relatively low loadings of fungal pellets. This behavior is commonly referred to as “Type III” adsorption and usually occurs when adsorbate-adsorbent interactions are small compared to the adsorbate-adsorbate interactions [40]. Such behavior is typical in the adsorption behavior of water to hydrophobic activated carbons where a limited number of surface functional groups act as primary adsorption centers for water and strong hydrogen bonding between water molecules creates “water clusters” containing multiple layers [41].

In this study, the observed Type III behavior removal is thought to be potentially attributed to factors including (1) radial fungal pellet growth during the immobilization experiment that caused an increase in the adsorbent-adsorbate (fungus-algae) interactions and number of potential immobilization sites; (2) auto-flocculation among algae cells due to extracellular secretions from the adsorbate causing strong adsorbate-adsorbate (algae-algae) interactions; and (3) adsorbent-facilitated flocculation originating from adsorbent (fungus) secretions that facilitated increased adsorbate-adsorbate interactions [16]. Several factors differentiate the results presented here from most other physical and chemical adsorption systems. First, the mass of fungal biomass considered here as the “adsorbent” is almost certainly not constant throughout the immobilization process, as evidenced by the visible filamentous hairs that grew and protruded from the pellet surfaces (Fig. 2B). In addition to the total mass of adsorbent, the total number of “active sites” for adsorption was similarly not constant through the experimental period. Future research studies could be directed towards the development of a theoretical model that can adequately describe the highly dynamic behavior of this biological immobilization system.

The pH of the medium is known to influence adsorption characteristics and its effect has been well documented in chemical and physical adsorption processes as well as in biological-sorption processes, including fungal-assisted algae immobilization [42, 43]. The relatively constant pH (~8.0) of the negative control (0 g fungi/g algae) flasks confirmed the low activity state of the microalgae *P. tricornutum* since pH usually increases under active autotrophic growth due to the utilization of inorganic carbon in the media [44]. Prior studies have reported both pH decreases [14, 45] and increases [46] during fungal-assisted immobilization of microalgae. Causes of the different results in the literature are not clear but may be strain specific and related to operational parameters such as fungal spore-assisted versus fungal pellet-assisted immobilization and/or the addition of glucose or other readily utilized carbon source during immobilization which might encourage secretion of organic acids by certain fungal strains [47]. Low pH has been reported to favor immobilization with certain strain combinations [45,47] but not others [46]. Choi *et al*. [46] noticed little impact of initial pH on the fungal pellet-assisted immobilization of *Synechocystis* sp. with *Aspergillus oryzae* pellets and suggested that the culture pH may be more important during fungal spore-assisted immobilization than fungal pellet-assisted immobilization, which is consistent with the present study.

The values of the attachment coefficient (*K_s_*) are lower than those reported by Bhattacharya *et al*. [15], who used lower fungal loadings than all but the lowest loading in the present study. The difference may be owed to the species of fungus and algae used in the respective studies and also differences in agitation speed or pellet size. The fungus utilized in that study [15] was *A. fumigatus* and the microalgae were *Chlorella pyrenoidosa*, *Chroococcus* sp1, and a native consortium isolated from a lake in New Delhi, India. The authors also reported a reduction in *K_s_* in response to similar agitation speeds used in the present study (150 rpm). Their observed *K_s_* value of 0.25 using a fungal loading of 0.2 g/g and agitation speed of 150 rpm was very similar to the value observed in this study (0.26) for a loading of 0.28 g/g and agitation speed of 150 rpm. However, the reported value of λ for these conditions was 0.1795 1/h^3^ while the value found in the present study was about 1000× less (1.36 × 10^-4^ 1/h^3^), indicating a significantly slower immobilization process. This may be due to differences in the fungal and algal strains utilized as well as other factors such as temperature (38°C versus 30°C in the present study) and the ionic strength of the medium since the f/2 medium used to grow *P. tricornutum* is high in salt concentration. Both kinetics models were generally less accurate for lower fungal loading treatments. One possible explanation for this could be that the impact of initial fungal pellet surface area became more important at lower loadings such that small differences in pellet size led to relatively higher changes in algae concentration. Future studies may consider reducing the pellet size tolerance, increasing biological replicates, and/or considering other pellet geometric measures (*e.g.*, roundness, smoothness, and/or density).

As demonstrated in this study, the use of equilibrium and kinetic modeling allows for quantitative comparison of strain combinations and operational parameters across studies and similar techniques are recommended for inclusion in future research studies in this area. The utilization of filamentous fungi as carriers and cell supports for other cell types is a rapidly expanding field. For instance, the fungal immobilization of yeast and mammalian cells was recently demonstrated for utilization in alcoholic beverage fermentations and cultivated meat production [48]. The insights and modeling techniques demonstrated here will help to provide industrially relevant benchmarks for the immobilization of other species of commercial or environmental interest and provide necessary data for future techno-economic and life cycle assessments.

## Figures and Tables

**Table 1:** Kinetic parameters determined for the pseudo-second order and Bhattacharya models.

Initial Fungi Loading	Pseudo-Second Order Model		Bhattacharya Model		

(g/g algae)	k (g/g•d)	R^2^	K_s_	λ (1/d^3^)	R^2^
0.28	0.93	0.67	0.26	1.88	0.84
0.64	4.1	0.88	0.35	2.52	0.91
1.30	18.9	0.99	0.51	3.85	0.98
2.53	-71.6	0.99	0.60	10.01	0.99

## References

[1] Mata TM, Martins AA, Caetano NS (2010). Microalgae for biodiesel production and other applications: a review. Renew. Sustain. Energy Rev..

[2] Moreno-Garrido I (2008). Microalgae immobilization: current techniques and uses. Bioresour. Technol..

[3] Caldwell GS, In-Na P, Hart R, Sharp E, Stefanova A, Pickersgill M (2021). Immobilising microalgae and cyanobacteria as biocomposites: new opportunities to intensify algae biotechnology and bioprocessing. Energies (Basel).

[4] Luo S, Wu X, Jiang H, Yu M, Liu Y, Min A (2019). Edible fungi-assisted harvesting system for efficient microalgae bioflocculation. Bioresour. Technol..

[5] Wang X, Yang C, Yu Y, Zhao Y (2022). In Situ 3D Bioprinting living photosynthetic scaffolds for autotrophic wound healing. Research (Wash DC).

[6] Zhou W, Min M, Hu B, Ma X, Liu Y, Wang Q, Shi J, Chen P, Ruan R (2013). Filamentous fungi assisted bio-flocculation: a novel alternative technique for harvesting heterotrophic and autotrophic microalgal cells. Sep. Purif. Technol..

[7] Zhang J, Hu B (2012). A novel method to harvest microalgae via co-culture of filamentous fungi to form cell pellets. Bioresour. Technol..

[8] Rajendran A, Hu B (2016). Mycoalgae biofilm: development of a novel platform technology using algae and fungal cultures. Biotechnol. Biofuels.

[9] Gultom S, Hu B (2013). Review of microalgae harvesting via Co-pelletization with filamentous fungus. Energies (Basel).

[10] Al-Hothaly KA, Adetutu EM, Taha M, Fabbri D, Lorenzetti C, Conti R (2015). Bio-harvesting and pyrolysis of the microalgae *Botryococcus braunii*. Bioresour. Technol..

[11] Muradov N, Taha M, Miranda AF, Wrede D, Kadali K, Gujar A (2015). Fungal-assisted algal flocculation: application in wastewater treatment and biofuel production. Biotechnol. Biofuels.

[12] Nasir NM, Bakar NSA, Lananan F, Abdul Hamid SH, Lam SS, Jusoh A (2015). Treatment of African catfish, *Clarias gariepinus* wastewater utilizing phytoremediation of microalgae, *Chlorella* sp. with *Aspergillus niger* bio-harvesting. Bioresour. Technol..

[13] Prajapati SK, Bhattacharya A, Kumar P, Malik A, Vijay VK (2016). A method for simultaneous bioflocculation and pretreatment of algal biomass targeting improved methane production. Green Chem..

[14] Bhattacharya A, Mathur M, Kumar P, Prajapati SK, Malik A (2017). A rapid method for fungal assisted algal flocculation: critical parameters & mechanism insights. Algal Res..

[15] Bhattacharya A, Malik A, Malik HK (2017). A mathematical model to describe the fungal assisted algal flocculation process. Bioresour. Technol..

[16] Bhattacharya A, Mathur M, Kumar P, Malik A (2019). Potential role of N-acetyl glucosamine in *Aspergillus fumigatus*-assisted *Chlorella pyrenoidosa* harvesting. Biotechnol. Biofuels.

[17] Miranda AF, Taha M, Wrede D, Morrison P, Ball AS, Stevenson T, Mouradov A (2015). Lipid production in association of filamentous fungi with genetically modified cyanobacterial cells. Biotechnol. Biofuels.

[18] Barzee TJ, Cao L, Pan Z, Zhang R (2021). Fungi for future foods. J. Future Foods.

[19] Zhou W, Cheng Y, Li Y, Wan Y, Liu Y, Lin X (2012). Novel fungal pelletization-assisted technology for algae harvesting and wastewater treatment. Appl. Biochem. Biotechnol..

[20] Zhao Y, Guo G, Sun S, Hu C, Liu J (2019). Co-pelletization of microalgae and fungi for efficient nutrient purification and biogas upgrading. Bioresour. Technol..

[21] Wrede D, Taha M, Miranda AF, Kadali K, Stevenson T, Ball AS (2014). Co-cultivation of fungal and microalgal cells as an efficient system for harvesting microalgal cells, lipid production and wastewater treatment. PLoS One.

[22] Silva A, Delerue-Matos C, Figueiredo SA, Freitas OM (2019). The use of algae and fungi for removal of pharmaceuticals by bioremediation and biosorption processes: a review. Water (Basel).

[23] Bowler C, Allen AE, Badger JH, Grimwood J, Jabbari K, Kuo A (2008). The *Phaeodactylum* genome reveals the evolutionary history of diatom genomes. Nature.

[24] Maheswari U, Mock T, Armbrust EV, Bowler C (2009). Update of the Diatom EST Database: a new tool for digital transcriptomics. Nucleic Acids Res..

[25] Hildebrand M, Davis AK, Smith SR, Traller JC, Abbriano R (2012). The place of diatoms in the biofuels industry. Biofuels.

[26] Burch AR, Franz AK (2016). Combined nitrogen limitation and hydrogen peroxide treatment enhances neutral lipid accumulation in the marine diatom *Phaeodactylum tricornutum*. Bioresour. Technol..

[27] Branco-Vieira M, Martin SS, Agurto C, Santos MA, Freitas MAV, Caetano NS (2017). Analyzing *Phaeodactylum tricornutum* lipid profile for biodiesel production. Energy Procedia.

[28] Burch AR, Yothers CW, Salemi MR, Phinney BS, Pandey P, Franz AK (2021). Quantitative label-free proteomics and biochemical analysis of *Phaeodactylum tricornutum* cultivation on dairy manure wastewater. J. Appl. Phycol..

[29] APHA, AWWA, WEF (2017). Standard Methods for the Examination of Water and Wastewater.

[30] Béchet Q, Laviale M, Arsapin N, Bonnefond H, Bernard O (2017). Modeling the impact of high temperatures on microalgal viability and photosynthetic activity. Biotechnol. Biofuels.

[31] Petruzzi L, Campaniello D, Speranza B, Corbo MR, Sinigaglia M, Bevilacqua A (2017). Thermal treatments for fruit and vegetable juices and beverages: a literature overview. Compr. Rev. Food Sci. Food Saf..

[32] Zepka LQ, Borsarelli CD, da Silva MAAP, Mercadante AZ (2009). Thermal degradation kinetics of carotenoids in a cashew apple juice model and its impact on the system color. J. Agric. Food Chem..

[33] Tseng RL, Wu FC (2008). Inferring the favorable adsorption level and the concurrent multi-stage process with the Freundlich constant. J. Hazard. Mater..

[34] Chaudhry SA, Khan TA, Ali I (2017). Zirconium oxide-coated sand based batch and column adsorptive removal of arsenic from water: isotherm, kinetic and thermodynamic studies. Egypt. J. Pet..

[35] Chaudhry SA, Khan TA, Ali I (2017). Equilibrium, kinetic and thermodynamic studies of Cr(VI) adsorption from aqueous solution onto manganese oxide coated sand grain (MOCSG). J. Mol. Liq..

[36] Ho YS (2004). Citation review of Lagergren kinetic rate equation on adsorption reactions. Scientometrics.

[37] Du ZY, Alvaro J, Hyden B, Zienkiewicz K, Benning N, Zienkiewicz A (2018). Enhancing oil production and harvest by combining the marine alga *Nannochloropsis oceanica* and the oleaginous fungus *Mortierella elongata*. Biotechnol. Biofuels.

[38] Hallab NJ, Bundy KJ, O'Connor K, Moses RL, Jacobs JJ (2001). Evaluation of metallic and polymeric biomaterial surface energy and surface roughness characteristics for directed cell adhesion. Tissue Eng..

[39] Kobayashi Y, Harada N, Nishimura Y, Saito T, Nakamura M, Fujiwara T (2014). Algae sense exact temperatures: small heat shock proteins are expressed at the survival threshold temperature in *Cyanidioschyzon merolae* and *Chlamydomonas reinhardtii*. Genome Biol. Evol..

[40] Keller JU, Staudt R (2005). Adsorption Isotherms. Gas Adsorption Equilibria: Experimental Methods and Adsorptive Isotherms.

[41] Brennan JK, Bandosz TJ, Thomson KT, Gubbins KE (2001). Water in porous carbons. Colloids and Surfaces A: Physicochem. Eng. Aspects.

[42] Zamalloa C, Gultom SO, Rajendran A, Hu B (2017). Ionic effects on microalgae harvest via microalgae-fungi co-pelletization. Biocatal. Agric. Biotechnol..

[43] Negm NA, Abd El Wahed MG, Hassan ARA, Abou Kana MTH (2018). Feasibility of metal adsorption using brown algae and fungi: effect of biosorbents structure on adsorption isotherm and kinetics. J. Mol. Liq..

[44] Uggetti E, Sialve B, Latrille E, Steyer JP (2014). Anaerobic digestate as substrate for microalgae culture: the role of ammonium concentration on the microalgae productivity. Bioresour. Technol..

[45] Xie S, Sun S, Dai SY, S. Yuan J (2013). Efficient coagulation of microalgae in cultures with filamentous fungi. Algal. Res..

[46] Choi YN, Cho HU, Utomo JC, Shin DY, Kim HK, Park JM (2016). Efficient harvesting of *Synechocystis* sp. PCC 6803 with filamentous fungal pellets. J. Appl. Phycol..

[47] Ogawa M, Garcia JM, Nitin N, Baar K, Block DE (2022). Assessing edible filamentous fungal carriers as cell supports for growth of yeast and cultivated meat. Foods.

